# A Simple Method for Improving the Odor of Iodoform

**Published:** 1881-05

**Authors:** J. Hanlo


Dr. J. Hanlo in the *Weekblatt van het Nederlandschr. Tydschrift von Genkeesunde*, gives a simple method for improving the odor of Iodoform. He claims that by the addition of any of the essential oils, preferably ol. menthæ pip., the disagreeable odor of iodoform may be entirely disguised. Of two preparations, the one composed of iodoform 2 grains and collodion 30 grains; and the other of iodoform 2 grains and vaseline 30 grains, the peculiar iodoform odor was completely covered by the addition of 6 drops of ol. menthæ pip.

